# Multi-Species Synbiotic Supplementation Enhances Gut Microbial Diversity, Increases Urolithin A and Butyrate Production, and Reduces Inflammation in Healthy Adults: A Randomized, Placebo-Controlled Trial

**DOI:** 10.3390/nu17172734

**Published:** 2025-08-23

**Authors:** Brooke A. Napier, Jessica R. Allegretti, Paul Feuerstadt, Colleen R. Kelly, Nicholas W. Van Hise, Ralf Jäger, Zain Kassam, Gregor Reid

**Affiliations:** 1 Seed Health, Inc., Venice, CA 90291, USA; brooke@seed.com; 2 Division of Gastroenterology, Hepatology, and Endoscopy, Brigham and Women’s Hospital, Harvard Medical School, Boston, MA 02115, USA; 3 Department of Digestive Diseases, Yale University School of Medicine, New Haven, CT 06510, USA; 4 Department of Gastroenterology, PACT Gastroenterology Center, Hamden, CT 06518, USA; 5 Metro Infectious Disease Consultants, Burr Ridge, IL 60527, USA; nvanhise@midcusa.com; 6 Increnovo LLC, Whitefish Bay, WI 53217, USA; 7 Canadian R&D Centre for Human Microbiome and Probiotics, Lawson Health Research Institute, London, ON N6A 4V2, Canada; gregor@uwo.ca; 8 Departments of Microbiology and Immunology and Surgery, Western University, London, ON N6A 3K7, Canada

**Keywords:** probiotic, prebiotic, short-chain fatty acids, urolithin, microbiome, synbiotic

## Abstract

**Background**: In healthy adults, probiotic supplementation alone does not increase Urolithin A (UroA) and rarely increases butyrate, both microbiome-derived metabolites that influence key biological functions involved in regulating gastrointestinal symptoms. Accordingly, this clinical trial evaluated key biological functions of a multi-species synbiotic with 24 probiotic strains and a polyphenol-based prebiotic using capsule-in-capsule delivery technology. **Methods**: We conducted a randomized, placebo-controlled trial among healthy participants (*n* = 32). Participants were administered a daily synbiotic (53.6 billion AFU multi-species probiotic and 400 mg Indian pomegranate extract; DS-01) or matching placebo for 91 days. Samples were obtained at baseline Day 0, and Days 7, 14, 49, and 91. Endpoints included changes in fecal microbiome composition, urinary UroA, fecal butyrate, serum CRP, and safety. **Results**: The synbiotic significantly increased alpha-diversity of *Bifidobacterium* and *Lactobacillus* spp. at all timepoints, including at end-of-study (Day 91, *p* < 0.0001) and increased native beneficial microbes. UroA production was significantly increased in the synbiotic arm at short-term (Day 7, 12-fold, *p* < 0.02) and long-term (Day 91, 49-fold, *p* < 0.001) timepoints. A higher proportion of synbiotic participants were capable of converting polyphenols into UroA (Day 91, 100% vs. 44.4%; *p* < 0.01). Mechanistically, synbiotic participants showed an increased abundance of *Lactobacillus* species involved in UroA precursor metabolism and UroA-producing *Gordonibacter* species. The synbiotic also significantly increased fecal butyrate levels (*p* < 0.03), and butyrate-producing species, in low-baseline butyrate producers, through Day 91, and was associated with reduced systemic inflammation. **Conclusions**: This multi-species synbiotic significantly increases diversity and abundance of key beneficial bacteria, enhances UroA production and butyrate levels, and is associated with lowered systemic inflammation. This is the first synbiotic to increase both UroA and butyrate.

## 1. Introduction

It is well documented that probiotics promote gastrointestinal (GI) health by modulating the microbiome, which in turn impacts gut barrier integrity, motility, gas production, immune function, and other key biological processes [1,2,3]. Synbiotics enable greater impact on biological function as they combine probiotics with prebiotics, dietary substrates that selectively promote the growth or activity of beneficial microorganisms [4]. Recently there has been an emergence of multi-species probiotics/synbiotics designed to enhance gut microbiota diversity, a microbial signature that has been consistently associated with improved overall health outcomes [5]. Clinical trials have shown that these multi-species synbiotics have meaningful benefits, including improving constipation in children [6], and maintaining microbiome diversity in adults with respiratory infections [7]. However, there is significant heterogeneity in formulations, and delivery technologies which impact survivability. Further, most clinical studies continue to use single strain probiotic/synbiotic interventions, limiting our understanding of how strain combinations perform in broad-spectrum formulations. Combining multiple strains in a single product may offer synergistic benefits or potentially introduce antagonistic interactions that could diminish efficacy. Accordingly, evaluating the mechanism of action and biological function of a multi-species synbiotic in its final formulation is critical.

Urolithin A (UroA) is a key microbiome-dependent metabolite that regulates fundamental biological functions. Preclinical data demonstrates UroA increases gut barrier integrity by increasing mucin, promotes mitochondrial health, decreases body weight via increased thermogenesis, and improves muscle function in aged animal models [8,9]. Clinically, UroA protects against age-related muscle mass decline via mitophagy, and promotes muscle strength and endurance in aging and young adults [10,11,12]. UroA production is dependent on both exposure to dietary polyphenols and gut microbiome composition and requires two metabolic steps. First, after consumption of dietary polyphenols that contain ellagitannins, microbes that express tannases including *L. rhamnosus*, *L. plantarum*, and *L. casei* metabolize ellagitannins into a precursor, ellagic acid [6]. Second, this precursor is subsequently metabolized by specific microbes (*Gordonibacter*, *Ellagibacter*, and *Entercloster* species) to produce UroA [8,9]. Despite the mechanistic understanding that both polyphenols and microbiome species are key for UroA production, there is a paucity of data demonstrating probiotics/synbiotics increase UroA in clinical trials.

Short-chain fatty acids (SCFA), including butyrate, propionate, and acetate, are key microbiome-mediated metabolites with broad biological function [13]. Among these, butyrate serves as a major energy source for colonic epithelial cells and plays a central role in GI, immune, and metabolic health [14]. Butyrate supports the integrity of the gut barrier by modulating tight junction proteins, and immune homeostasis by inhibiting proinflammatory cytokine production and regulating T-cell differentiation [15,16]. Additionally, butyrate stimulates the release of glucagon-like peptide-1 (GLP-1) from intestinal L-cells and acts via the vagus nerve, a mechanism with implications for obesity and type 2 diabetes [17]. Clinically, butyrate may alleviate bloating by enhancing GI motility and decreasing visceral hypersensitivity via barrier function optimization [18,19]. Microbially, beneficial bacteria, such as certain *Bifidobacterium* and *Lactobacillus,* have demonstrated the ability to produce key metabolites (e.g., lactate, acetate) that cross-feed native butyrate-producing microbes, in turn, increasing butyrate [20]. Biologically, probiotics/synbiotics are unlikely to increase butyrate-levels among healthy adults with optimal baseline levels of butyrate. This is supported by a systematic review which reports probiotic supplementation in healthy adults did not reliably alter fecal microbiota composition or significantly increase butyrate levels [21]. However, individuals with lower baseline levels of butyrate may experience a measurable increase in butyrate after probiotic/synbiotic intervention as they are not in homeostasis. For example, a clinical trial involving healthy adults who consumed *Bifidobacterium bifidum* Bb reported that changes in fecal butyrate concentrations were dependent on participants’ baseline SCFA levels [22]. Given the biological benefit of butyrate, elucidating the impact of multi-species synbiotics on butyrate in clinical trials, especially among those with low baseline butyrate, is important.

Accordingly, this clinical trial evaluated key biological functions of a multi-species synbiotic with 24 probiotic strains and a polyphenol-based prebiotic using capsule-in-capsule delivery technology.

## 2. Methods

### 2.1. Clinical Trial Design

We conducted a randomized, placebo-controlled, double-blind, parallel-group clinical trial among healthy adults (KGK Science Inc., London, ON, Canada). The study was approved by an Institutional Review Board (Advarra IRB, Columbia, MD, USA) in accordance with the Declaration of Helsinki regarding the ethics of human participant research. All participants signed written consent forms to participate in this study. Participants ingested 2 capsules daily of a multi-species synbiotic or a matching placebo for 91 days. The primary outcome was change in microbiota composition at Day 91 as assessed by whole genome shotgun sequencing. Secondary outcomes included changes in intestinal butyrate production, as assessed by metabolomics on whole stool samples; UroA production as measured in urine; change in systemic inflammation as assessed by serum C-reactive protein (CRP) levels; and safety, as assessed by vital signs, clinical chemistry and hematology.

**Figure 1:**
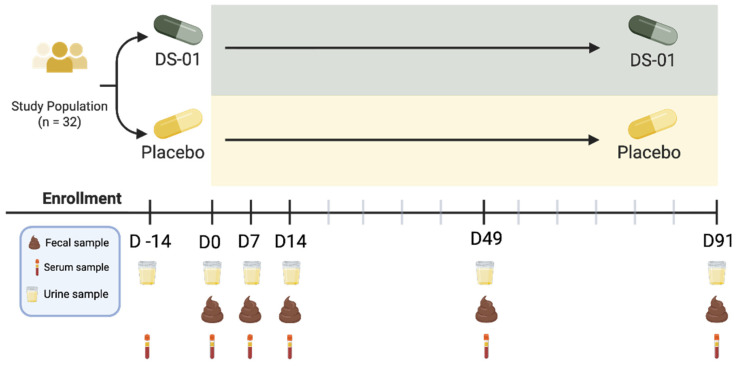
**Overview of clinical trial design.** Stool collection for fecal metagenomics and metabolomics. Urine collection for Urolithin A analysis on Day 0, 7, 14, 49 and 91, and pregnancy test for female participants on Day −14, 0 and 91. 12-h fasted blood samples for CRP analysis on Day 0, 7, 14, 49 and 91; safety markers on Day −14 and 91. Note: Study materials were dispensed on Day 0, 7, 14, and 49; empty study material packaging was returned on Day 7, 14, 49, and 91.

Figure 1 shows the clinical trial overview. Baseline 3-day food record, stool, urine, blood and vital signs (resting HR, BP, weight) were collected. Participants repeated this procedure after Day 7, 14, 49 and 91.

### 2.2. Study Participants

Participants were recruited through email, posting, and/or publishing advertisement leaflets in online and local print locations. Those who expressed interest in participating in the study were consulted via email or phone to determine their general eligibility. Eligibility was determined according to inclusion and exclusion criteria (Table 1).

**Figure 2:**
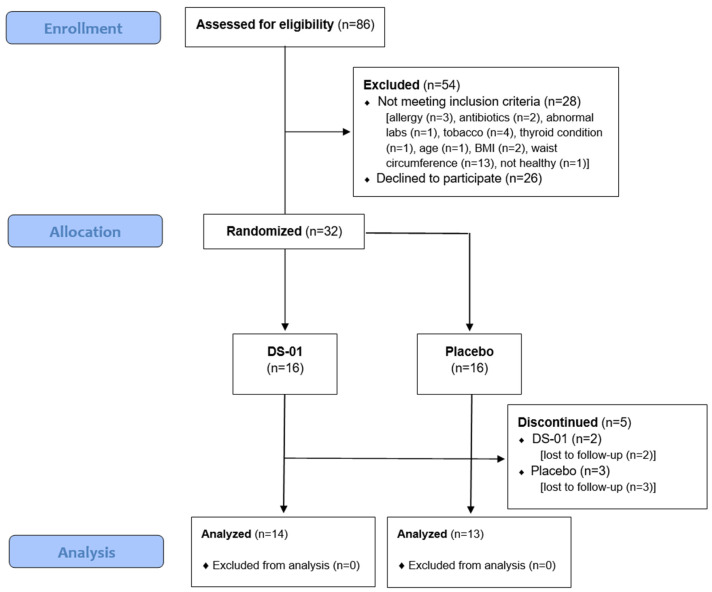
Consolidated Standards of Reporting Trials (CONSORT) flow chart for recruitment, allocation, and analysis of the study arms.

Figure 2 shows a Consolidated Standards of Reporting Trials (CONSORT) diagram. A total of 86 interested individuals replied to recruitment and had eligibility examined, of which 32 met screening eligibility requirements. Participants were consented to participate and were randomized 1:1 using https://www.random.org (accessed on 23 July 2025) into their respective trial arms. 5 participants were lost due to follow-up (Placebo: *n* = 3; DS-01: *n* = 2). Statistical analysis was performed on 27 participants who completed the study.

### 2.3. Screening

Participants who met preliminary eligibility criteria were invited to an in-person session where they obtained information on the study protocols and provided written informed consent. Individuals meeting inclusion criteria completed a health assessment that included measurement of height, weight, resting heart rate (HR), resting blood pressure (BP), and fasting blood evaluation. Blood samples were used for screening to assess eligibility. Finally, they were provided with instructions on completing food logs using an online food record application (Libero, v6.11, Nutritics, Dublin, Ireland). Participants were instructed to record a detailed food and energy-containing fluid intake for 4 days (3 weekdays and 1 weekend day) before the second, fourth, and sixth visits to the laboratory. They were instructed to avoid any foods or beverages containing live bacteria throughout the duration of the trial. This included probiotic supplements and any food, capsule, or orally administered product containing live bacteria or yeast (e.g., *Lactobacillus*, *Bifidobacteria*, *Bacillus*, *Saccharomyces*) and prebiotic supplements. Restricted foods included yogurt; naturally fermented cabbage products (e.g., sauerkraut, kimchi); naturally fermented pickles, cucumbers, and other vegetables; fermented soy products (e.g., tempeh, miso, natto); uncooked buttermilk products; soft cheeses and live culture cheeses (e.g., blue cheese, gouda, goat cheese); cold or uncooked sausage products; and any foods marketed as “Probiotic” or “Live Culture.” Restricted beverages included kefir, kombucha, apple cider vinegar, lambic beers, sour ales, wheat beers, and any beverage labeled as probiotic. The food records were reviewed by trained staff at the visits and participants were counseled with dietary suggestions as required. Participants were asked to replicate this exclusionary diet prior to and following each testing session to standardize dietary intake across sessions.

### 2.4. Multi-Species Synbiotic Intervention

Participants were randomized to ingest 2 capsules of a multi-species synbiotic with novel capsule-in-capsule delivery technology (ViaCap) totaling 400 mg of prebiotic Indian pomegranate (*Punica granatum*, >40% polyphenols) extract and 53.6 billion AFU of a multi-species probiotic containing 24 different bacteria strains: *Bifidobacterium longum* SD-BB536-JP, *Bifidobacterium breve* SD-BR3-IT, *Lactiplantibacillus plantarum* SD-LP1-IT, *Lacticaseibacillus rhamnosus* SD-LR6-IT, *Lacticaseibacillus rhamnosus* HRVD113-US, *Bifidobacterium infantis* SD-M63-JP, *Bifidobacterium lactis* SD-BS5-IT, *Bifidobacterium lactis* HRVD524-US, *Lactobacillus crispatus* SD-LCR01-IT, *Lacticaseibacillus casei* HRVD300-US, *Bifidobacterium breve* HRVD521-US, *Bifidobacterium longum* HRVD90b-US, *Bifidobacterium lactis* SD150-BE, *Limosilactobacillus fermentum* SD-LF8-IT, *Lacticaseibacillus rhamnosus* SD-GG-BE, *Limosilactobacillus reuteri* RD830-FR, *Ligilactobacillus salivarius* SD-LS1-IT, *Bifidobacterium lactis* SD-CECT8145-SP, *Bifidobacterium longum* SD-CECT7347-SP, *Lacticaseibacillus casei* SD-CECT9104-SP, *Lactiplantibacillus plantarum* SD-LPLDL-UK, *Bifidobacterium lactis* SD-MB2409-IT, *Bifidobacterium adolescenti*s SD-BA5-IT, and *Limosilactobacillus reuteri* SD-LRE2-IT (DS-01, Seed Health, Inc., Venice, CA, USA) or a matching placebo (rice flour). The multi-species synbiotic was designed using a bioinformatics-driven selection process that identifies strains based on genomic redundancy, pan-genomic coverage, functional profiles, and microbial networks. The formulation approach was to maximize microbial genomic diversity and total/unique microbial genes to optimize efficacy across a diverse population. From a functional perspective, the consortium is supported by mechanistic and preclinical evidence with its ability to (i) supply lactate- and acetate-producing taxa that cross-feed endogenous butyrate producers, and (ii) pair these microbes with ellagitannin substrates that support UroA production by native converters. Although not every strain is intended to directly produce UroA or butyrate, the formulation facilitates these outcomes through networked ecological functions. Notably, the identical final product was previously evaluated in a gut model simulating the luminal and mucosal colonic environment with an inoculated human microbiome. In this experimental model, the muti-species synbiotic restored and enhanced SCFA output after perturbation, including significantly increasing butyrate levels compared to controls. This *in vitro* evidence provides preclinical support for the consortium used in the clinical trial [23]. Participants were instructed to ingest the capsules once per day in the morning right before breakfast on an empty stomach following the baseline session (Day 0) and continuing each day throughout the study for 91 days. Participants were asked to save all unused and open packages and return them to the lab for a determination of compliance. If a dose was missed, participants were instructed to continue supplementation (2 capsules) the next day.

### 2.5. Vitals Signs

Height and weight were obtained. Resting HR and BP were determined in an upright, seated position after a five-minute rest period. Palpation of the radial artery was used to calculate resting heart rate according to standard procedures.

### 2.6. Blood Sampling

An appropriately trained and qualified phlebotomist performed the venipuncture procedure to collect the necessary blood samples. Participants were placed in a comfortable seated position with their desired arm, at the phlebotomist’s discretion, fully extended and supported with a pillow. A tourniquet was applied 3–4 inches above the elbow with the participants opening and closing their fist a few times to allow the phlebotomist to manually determine the approximate size, depth, and location of the vein. Following the site of the venipuncture being appropriately sterilized, the phlebotomist collected the sample using the vacutainer system according to the relevant laboratory order requirement.

### 2.7. Stool Sample Collection and Fecal Metabolomic and Metagenomic Analysis

Participants were given a stool collection kit containing materials required to collect their stool sample along with ice-packs. They received both verbal and written instructions to ensure standardized collection procedures, including guidance on timing, handling and storage. Participants were also instructed to replicate their diet to minimize dietary-driven variability in stool composition. The sample was frozen immediately after collection and transported, on ice, within 2 days of collection. For fecal butyrate analysis, the fecal matter was analyzed for butyrate production via mass spectrometry (GC-MS). For fecal metagenomics, stool DNA extraction, library preparation and sequencing were performed at the University of Maryland Genomics Core (Rockville, MD, USA). Taxonomic profiling was performed using the Xtree tool (derived from SHOGUN-UTree, Version 2.00a) to map shotgun sequencing reads against custom genome databases. A species-level (~95% ANI) database consisting of all GTDB R207 representatives and the resulting count tables were used as input for calculations of diversity. Synbiotic strain abundance was observed together with >600 additional genomes from the genera comprising the synbiotic consortium to examine specificity. 7106 genomes meeting criteria (in GTDB R220; in Refseq; CheckM2 completeness ≥90%, contamination ≤5%; in genera *Bifidobacterium*, *Lacticaseibacillus*, *Lactiplantibacillus*, *Lactobacillus*, *Limosilactobacillus*, and *Ligilactobacillus*) were clustered with 24 synbiotic strain genomes at 99.2% ANI, into 643 strain-level groups total, represented by one genome each. Representatives consisted of independent synbiotic strain genomes, 384 *lactobacilli* strains, and 241 *Bifidobacterium* strains. Synbiotic strain genomes were marked present based on 15% and 25% unique and total k-mers present, or if >90% total-kmers were found. Reads from genomes were divided by total sample reads to calculate relative abundance.

### 2.8. Urine Collection

The first void on the morning of the study visits were collected and changes in UroA was assessed via mass spectrometry (LC-MS/MS) (Arome Science Inc., Farmington, CT, USA). Urine samples were thawed on ice, and 600 µL was aliquoted into a polypropylene 96-well plate (Eppendorf, Enfield, CT, USA). A 100 µL volume of dilution solvent (Sulfachloropyridazine and Sulfamethazine at 25 µM in HPLC-grade water) was added to each well, and the plate was sonicated on ice for 20 min. Homogenized samples were transferred to an Oasis HLB µElution Plate (Waters Corporation, Milford, MA, USA) using a TOMTEC Quadra liquid handler (Tomtec Inc., Hamden, CT, USA), and conditioned with LC-MS grade methanol and water. Samples were then filtered and eluted using sequential organic and aqueous washes. The eluted samples were lyophilized in a thermally controlled vacuum centrifuge (Genevac HT-4x, Genevac Ltd., Ipswich, United Kingdom) and subsequently resuspended in 100 µL of resuspension buffer (Sulfamethizole and Sulfadimethoxine at 25 µM in a 1:1 methanol:water solution). Resuspended samples were sonicated on ice for 20 min and centrifuged at 14,000 rpm for 10 min. Chromatographic separation was performed using a Vanquish UPLC system equipped with a Kinetex 1.7 µm C18 100 Å column (Phenomenex, Torrance, CA, USA), maintained at 40 °C with a flow rate of 0.4 mL/min. The mobile phases consisted of water with 0.1% formic acid (A) and acetonitrile with 0.1% formic acid (B). The gradient program was as follows: 0–0.5 min, 5% B; 0.5–5.0 min, 60% B; 5.0–5.1 min, 100% B; 5.1–6.1 min, 100% B; 6.1–6.2 min, 5% B, held until 8.0 min. Mass spectrometry analysis was conducted on an Orbitrap mass spectrometer (Thermo Fisher Scientific Inc., Waltham, MA, USA) equipped with a H-ESI probe source. External mass calibration was performed prior to acquisition, achieving a mass accuracy of less than 1 ppm. Quantification was conducted using Thermo Xcalibur software (Thermo Fisher Scientific Inc., Waltham, USA, Version 4.6.67.17) with external calibration. Methanol (MeOH, HPLC grade), Acetonitrile (ACN, HPLC grade), Water with 0.1% Acetonitrile (Optima LC-MS), Water (H_2_O, HPLC grade), Ellagic acid, and UroA were purchased from Fisher Chemicals (Bridgewater, NJ, USA). Sulfamethizole, Sulfadimethoxine, Sulfamethazine, Sulfachloropyridazine, and Water with 0.1% Formic acid (Optima, LC-MS) were purchased from Thermo Scientific (Waltham, MA, USA).

### 2.9. Safety and Adverse Events

Safety was evaluated by monitoring vital signs (BP and HR) throughout the study, assessing the frequency and severity of adverse events, and analyzing changes in blood parameters. Blood safety parameters included alanine aminotransferase (ALT), aspartate transaminase (AST), total bilirubin, creatinine, electrolytes (Na, K, Cl), hemoglobin A1c (HbA1c), fasting glucose, estimated glomerular filtration rate (eGFR), white blood cell (WBC) count with differential (neutrophils, lymphocytes, monocytes, eosinophils, basophils), red blood cell (RBC) count, hemoglobin, hematocrit, platelet count, RBC indices (mean corpuscular volume (MCV), mean corpuscular hemoglobin (MCH), mean corpuscular hemoglobin concentration (MCHC), mean platelet volume (MPV), and red cell distribution width (RDW). Adverse events and changes in blood parameters were compared from screening (Day -14) to the final visit (Day 91).

### 2.10. Statistical Analysis

All data was analyzed in R (version 4.4.3, [24]). Linear mixed effects models were run using package ‘lme4′ (version 1.1–35.3) or ‘nlme’ (version 3.1–167). Analysis of the Urolithin A continuous valued data, fecal alpha-diversity of *Bifidobacterium* and *Lactobacillus*, fecal synbiotic strain relative abundance, fecal *lactobacilli* relative abundance, and change in fecal *Lactobacillus plantarum, L. casei, L. rhamnosus* relative abundance analyses were completed using linear mixed effects models with time point (discrete), study product, and their interaction as fixed effects, and subject ID as a random effect. For alpha-diversity of *Bifidobacterium* and *Lactobacillus* and synbiotic strain abundance, fold-change is calculated as [synbiotic/placebo]. For alpha-diversity of *Bifidobacterium* and *Lactobacillus,* percent difference is calculated as (synbiotic − placebo)/placebo. The percent difference gives us a measure of the difference in the synbiotic relative to the placebo. For synbiotic strain abundance, the percent difference is calculated using the geometric means to account for the log-transformations used on these data and are relative to the average of synbiotic and placebo; thus, calculated as (synbiotic − placebo)/[(synbiotic + placebo)/2]. Categorical analysis of UroA producers was completed using Fisher’s Exact Test (package ‘stats’, version 4.6.0). Low-baseline fecal butyrate analysis was completed using a linear mixed effects model with baseline split (low vs. high baseline fecal butyrate), Day (continuous), and their interaction as fixed effects and participant ID as a random effect. Change in CRP vs. change in synbiotic species relative abundance analysis was completed using a linear mixed model with change in synbiotic species relative abundance as the fixed effect and subject ID and visit as random effects. For all linear mixed effects models, violations of model assumptions were assessed visually and using the Shapiro–Wilk test for normality. Adjustments to models were made in the case of violations, including variable transformations and heterogeneous variances models (nlme).

## 3. Results

### 3.1. Participant Demographics

Table 2 shows participant demographics at baseline.

### 3.2. Microbiome Profile

**Figure 3:**
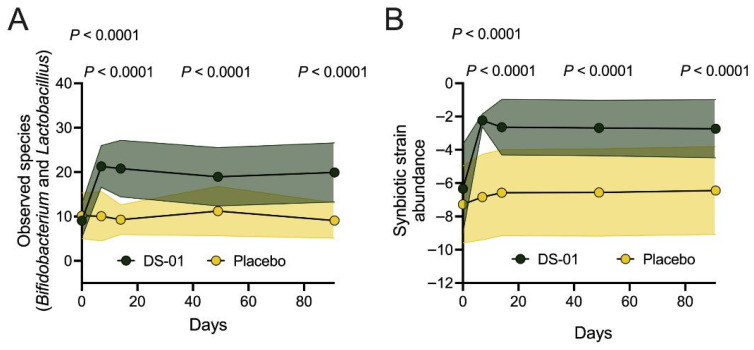
**Changes in fecal microbiome composition in healthy adults.** Healthy adults underwent daily administration of a multi-species synbiotic or placebo: (**A**) Observed species over time between arms, measured by alpha-diversity of beneficial microbes (combined *Bifidobacterium* and *Lactobacillus* species), as measured through taxonomic profiling of stool metagenomes. (**B**) Synbiotic strain abundance, or the summed abundance of all detected synbiotic strains within the stool [total combined abundance of all synbiotic strain genomes (at 99.2% ANI)], measured with the same method. Standard deviation of the arm was plotted with error polygons. Significance was measured between arms using linear mixed modeling.

Microbiome profiles of key beneficial microbes were characterized using alpha-diversity of *Bifidobacterium* and *Lactobacillus* species, the abundance of synbiotic strains, and abundance of native beneficial species. The alpha-diversity of *Bifidobacterium* and *Lactobacillus* species was significantly higher in the multi-species synbiotic arm compared to placebo at Day 7 (2.1-fold-change, 111% difference, *p* < 0.0001), Day 14 (2.2-fold-change, 124% difference, *p* < 0.0001), Day 49 (1.7-fold-change, 68% difference, *p* < 0.0001), and Day 91 (2.2-fold-change, 120% difference, *p* < 0.0001) (Figure 3A). More specifically, the multi-species synbiotic arm had a significant increase in the abundance of synbiotic strains, compared to placebo, at Day 7 (205-fold-change, 480% difference, *p* < 0.0001), at Day 14 (76-fold-change, 366% difference, *p* < 0.0001), at Day 49 (74-fold-change, 338% difference, *p* < 0.0001), and Day 91 (114-fold-change, 375% difference, *p* < 0.0001) (Figure 3B). The significantly increased synbiotic strains include *Bifidobacterium breve* SD-BR3-IT (Day 91; 8-fold-change, 691% difference, *p* < 0.0001) and *Lactiplantibacillus plantarum* SD-LP1-IT (Day 91; 204-fold-change, 20328% difference, *p* < 0.0001), two strains previously found to increase gut barrier function, and decrease bloating and gas [25].

Lastly, we find in healthy adults, the synbiotic increased the abundance of key native beneficial bacteria at Day 91 (end-of-study), compared to placebo, including species from known butyrate-producing genera *Roseburia* (2.6-fold-change, 163% difference, *p* < 0.01), *Butyribacter* (1.8-fold-change, 82% difference, *p* < 0.05), and *Blautia A* (1.7-fold-change, 71% difference, *p* < 0.05).

### 3.3. Urolithin a Production and Urolithin-Producing Population Dynamics

**Figure 4:**
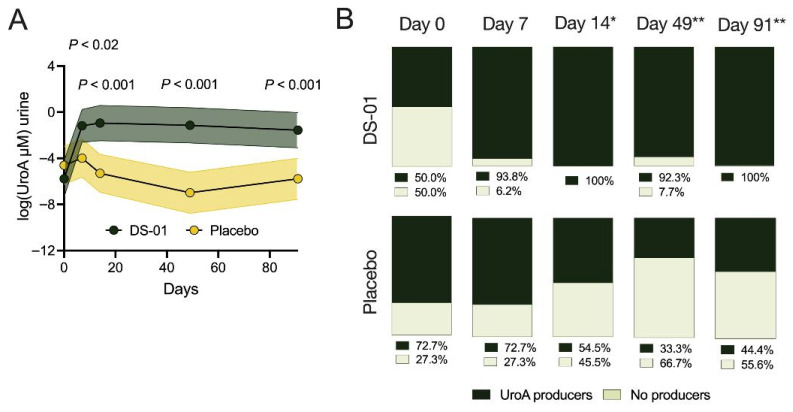
Daily supplementation with multi-species synbiotic increases UroA production and the proportion of UroA-producers in the population. Healthy adults underwent daily administration of a multi-species synbiotic or placebo: (**A**) Urinary UroA (μM) measured throughout the study. (**B**) The proportion of participants with detectable levels of UroA in the urine (UroA-producers) or without UroA detected in the urine (no producers) throughout each timepoint in the study. For A, standard deviation of the arm was plotted with error polygons. For (**A**), significance was measured between arms using linear mixed-effects modeling. For (**B**), significance was measured between arms using Fisher’s exact test (*, *p* < 0.05; **, *p* < 0.01).

There was a significant increase in urinary UroA production in the multi-species synbiotic arm, compared to placebo, at short-term endpoints (Day 7, 12-fold-change, 1141% difference, *p* < 0.02; Day 14, 60-fold-change, 5947% difference, *p* < 0.001,) and long-term (Day 49, 403-fold-change, 40,162% difference, and Day 91, 49-fold-change, 4829% difference, both *p* < 0.001) timepoints. (Figure 4A). Specifically, the multi-species synbiotic rapidly increased UroA production ~100-fold over baseline (Day 7, *p* < 0.0001), and retained this increase throughout the study (Figure 4A). At baseline (Day 0), 50.0% of the participants within the multi-species synbiotic arm and 72.7% of the participants within the placebo arm produced detectable amounts of urinary UroA, termed “UroA producers” (Figure 4B). By Day 14, the proportion of UroA producers in the multi-species synbiotic arm was significantly higher than the placebo arm (100% vs. 54.5%; *p* < 0.02), and this increase continued through Day 49 (92.3% vs. 33.3%; *p* < 0.01) and at the end of the study (Day 91, 100% vs. 44.4%; *p* < 0.01) (Figure 4B).

**Figure 5:**
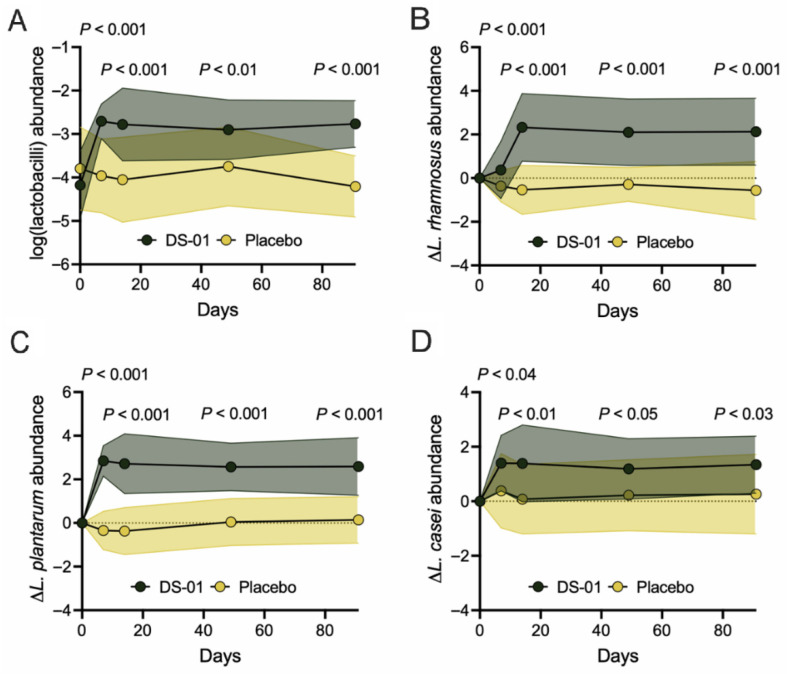
**Daily supplementation with multi-species synbiotic increases ellagic acid-producing *Lactobacillus* species abundance.** Healthy adults underwent daily administration of a multi-species synbiotic or placebo: (**A**) The relative abundance of lactobacilli, defined here as the summed relative abundance of species within the genera *Lactobacillus, Ligilactobacillus*, *Lacticaseibacillus*, *Lactiplantibacillus*, and *Limosilactobacillus*, as measured through taxonomic profiling of stool metagenomes. The change in relative abundance of (**B**) *Lactobacillus rhamnosus,* (**C**) *Lactobacillus plantarum,* and (**D**) *Lacticaseibacillus casei* measured with the same method. For (**A**–**D**), standard deviation of the arm was plotted with error polygons and significance was measured between arms using linear mixed-effects modeling.

Mechanistically, some *Lactobacillus* species have been shown to metabolize ellagitannins into UroA precursor, ellagic acid. Microbiome analysis demonstrated the mean overall abundance of all detected lactobacilli was significantly higher in participants in the multi-species synbiotic arm compared to those in the placebo at all time points (Day 7: 17-fold-change, 234% difference, *p* < 0.001; Day 14: 17-fold-change, 231% difference, *p* < 0.001; Day 49: 7-fold-change, 179% difference, *p* < 0.01; Day 91: 28-fold-change, 259% difference, *p* < 0.001) (Figure 5A). There are three *Lactobacillus* species within the multi-species synbiotic that have been shown *in vitro* to produce ellagic acid from ellagitannins [*Lacticaseibacillus rhamnosus*, *Lactiplantibacillus plantarum*, and *Lacticaseibacillus casei*] [6,7]. Specifically, all of these species were enriched in the multi-species synbiotic arm compared to placebo at all time points: *L. rhamnosus* (Day 7, 539-fold-change, 53,840% difference, *p* < 0.001; Day 14, 728-fold-change, 72,667% difference, *p* < 0.001; Day 49, 248-fold-change, 24,663% difference, *p* < 0.001; Day 91, 485-fold-change, 48,377% difference, *p* < 0.001), *L*. *plantarum* (Day 7, 1564-fold-change, 156,303% difference, *p* < 0.001; Day 14, 1224-fold-change, 122,268% difference, *p* < 0.001; Day 49, 334-fold-change, 33,312% difference, *p* < 0.001; Day 91, 280-fold-change, 27,922% difference, *p* < 0.001), and *L*. *casei* (Day 7, 10-fold-change, 932% difference, *p* < 0.04; Day 14, 20-fold-change, 1939% difference, *p* < 0.01; Day 49, 9-fold-change, 826% difference, *p* < 0.05; Day 91, 12-fold-change, 1112% difference, *p* < 0.03) (Figure 5B–D).

Notably, the abundance of UroA-producing *Gordonibacter* species was significantly increased in participants in the synbiotic arm at Day 7 (1.6-fold-change, 63% difference, *p* < 0.05) and Day 14 (1.8-fold-change, 80% difference, *p* < 0.02), compared to baseline. This was not seen in the placebo arm. Additionally, within our study, *Gordonibacter* abundance in the stool was positively correlated with UroA among multi-species synbiotic arm participants (*p* < 0.0001).

### 3.4. Butyrate

**Figure 6:**
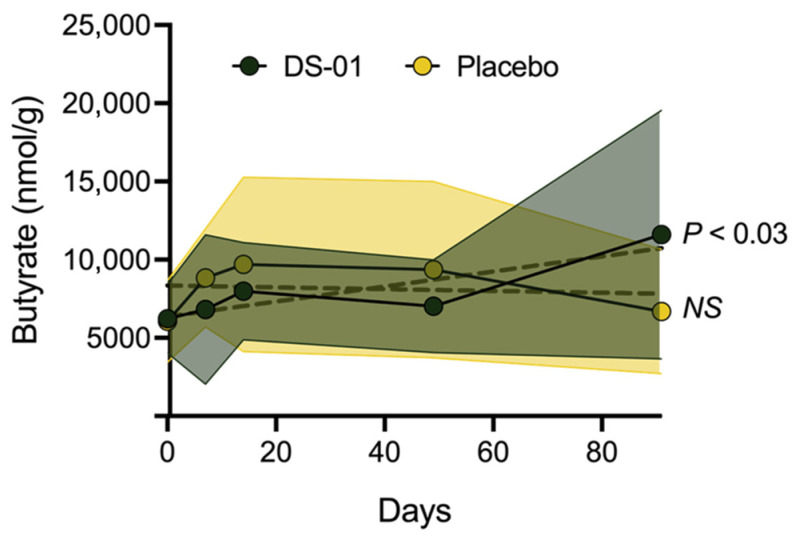
**Multi-species synbiotic supplementation significantly increases fecal butyrate levels.** Participant stool samples were collected and butyrate was measured via GC-MS. Analysis was conducted in a low-baseline butyrate population (lower than median baseline butyrate levels). Standard deviation of arm is plotted with error polygons. Significance is measured using linear mixed modeling.

As previously outlined, since clinical trials have shown that specific probiotics are most effective in individuals with low baseline butyrate levels, we evaluated the multi-species synbiotic in this key population [22,26,27]. Overall, DS-01 significantly increased fecal butyrate through Day 91 (*p* < 0.03) among low-baseline butyrate producers (defined by a lower-than-median butyrate at baseline). Specifically, there was a 66% increase in fecal butyrate in the synbiotic arm, whereas the placebo arm did not change (Figure 6).

Importantly, the synbiotic increased the abundance of native butyrate-producing bacteria in participants with low-baseline butyrate levels over time, including species from known butyrate-producing genera *Butyribacter* (*p* < 0.05; 78% increase from baseline). There was no significant increase of *Butyribacter* in the low-baseline butyrate population within the placebo arm.

### 3.5. Inflammation

Many strains within the multi-species synbiotic have been shown *in vitro* and in clinical trials to regulate inflammation [28,29,30,31,32]. CRP is a primary marker of systemic inflammation and widely recognized as a predictor of cardiometabolic and chronic disease risk. Participants within this trial had a healthy range of serum CRP (mean = 1.2 mg/L; SD = 1.43 mg/L). We assessed the impact of this multi-species synbiotic on optimizing inflammatory homeostasis within a healthy range by measuring the impact of multi-species synbiotic strain abundance on change of CRP.

**Figure 7:**
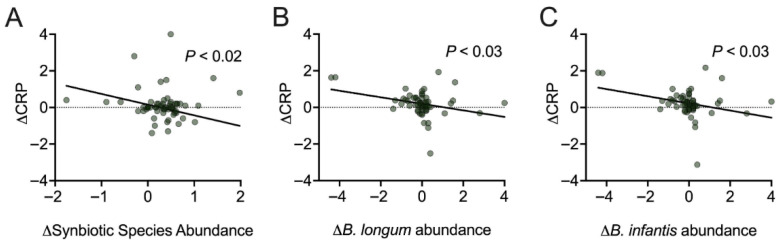
An increase in abundance of multi-species synbiotic species in the stool is associated with a reduction in serum CRP. Participants’ blood and stool samples were collected. Within the multi-species synbiotic arm, (**A**) the change in serum CRP plotted against the change in multi-species synbiotic species abundance (the log10 value of the summed abundance of all *Lactobacillus* and *Bifidobacterium* species found within synbiotic) within the stool. (**B**) The change in CRP plotted against the change in abundance of *Bifidobacterium longum* and (**C**) *Bifidobacterium infantis*. Significance is measured via linear mixed modeling.

Overall, we found that with the increased abundance of multi-species synbiotic species through Day 91, there was an associated reduction in serum CRP (*p* < 0.02) (Figure 7); however, there was no change in the placebo arm. More specifically, there was a reduction in serum CRP associated with the increased abundance of 2 synbiotic species, *B. longum* (*p* < 0.03) and *B. infantis* (*p* < 0.03). The multi-species synbiotic effect on inflammation resulted in a decrease of 0.44 mg/L of serum CRP among participants with a 10-fold increase in synbiotic species abundance compared to baseline.

### 3.6. Safety

The multi-species synbiotic was well-tolerated, and none of the participants reported adverse events in either study arm. No clinically meaningful effects were observed for changes in the clinical chemistry and hematology makers between study arms. Additionally, observed changes from baseline within each arm were deemed to be normal fluctuations not specific to any product. All values were within the normal range, confirming that the participants were healthy (Table 3).

## 4. Discussion

This randomized, placebo-controlled clinical trial provides compelling evidence that supplementation with a multi-species synbiotic formulation, containing 24 probiotic strains and a polyphenol-based prebiotic using a capsule-in-capsule delivery system [33], has meaningful impact on microbiome composition and microbiome-dependent biology that drives GI health. Major strengths of this study are the inclusion of a healthy adult population to increase generalizability of results, multiple sample collections across time, and deep biological interrogation using ultra-deep sequencing with 100 M read depth in addition to metabolomic characterization. Overall, there were five key findings.

First, the multi-species synbiotic increased microbial alpha-diversity of *Bifidobacterium* and *Lactobacillus* species, key beneficial microbes. The multi-species synbiotic effect was both rapid and persistent, with significant increase at Day 7 (*p* < 0.0001) and Day 91 (*p* < 0.0001). Broad alpha-diversity of the gut microbiome has been reported to decline with aging, a trend linked to reduced gut resilience, increased inflammation, and compromised health. Several studies have shown that elderly populations tend to exhibit lower microbiome diversity compared to younger adults, although this can vary with diet, medication use, and overall health status [34,35]. The observed increase in diversity with synbiotic supplementation in our study suggests a role in maintaining or restoring microbial diversity during healthy aging, and potentially contributing to GI and systemic health. Importantly, in our study we find the multi-species synbiotic increases *Bifidobacterium* and *Lactobacillus* species, which have been shown to decrease bloating and gas, most likely through metabolizing gas-producing dietary substrates (lactose and resistant starches) and competitively inhibiting gas-producing microbes. Additionally, there was an increase in species from known butyrate-producing genera, including *Roseburia*, *Butyribacter*, and *Blautia,* which have well-established biological benefits, including: promoting barrier function to decrease visceral hypersensitivity in bloating, regulating motility pathways that impact constipation and bloating, impacting overall immune function, and mediating healthy aging. Overall, this multi-species synbiotic increases diversity and native butyrate-producers that regulate biological function that impacts healthy aging and GI symptoms.

Second, the multi-species synbiotic increased UroA, a key microbiome-dependent metabolite that drives various aspects of human health. These data show that supplementation with this multi-species synbiotic significantly increases the production of UroA and converts 100% of the population into UroA-producers, or participants that can metabolize ellagitannins into UroA. Previously, it was shown that 24 h after dietary intervention with pomegranate juice, 67% of the population was converted into UroA-producers (via quantification of UroA-glucuronide in the serum) [36]. Our study demonstrates 100% conversion of healthy adults into UroA-producers, suggesting the potential superiority of multi-species synbiotic compared to dietary intervention alone. Mechanistically, this specific multi-species synbiotic enhanced the abundance of 3 known *Lactobacillus* species that can metabolize ellagitannins into UroA precursors. Further, UroA-producing *Gordonibacter* species abundance increases in multi-species synbiotic use, and UroA production was positively correlated with the enriched abundance of *Gordonibacter* species, similar to the results of a study of adults who consumed dietary ellagitannins [37]. These data suggest the multi-species synbiotic is bolstering an ecological niche for *Gordonibacter* species, and that *Gordonibacter* species may be among the important microbes for the synbiotic-dependent increase in UroA production. While these mechanisms are supported by prior literature, they were not directly tested in this study and may benefit from further validation. Overall, this multi-species synbiotic is the first to confer an increase in UroA, a key microbiome metabolite shown to impact healthy aging and GI function.

Third, the multi-species synbiotic significantly increased fecal butyrate and butyrate-producing species among individuals with non-optimal butyrate levels. Individuals with optimal butyrate levels are unlikely to increase their butyrate to supra-physiological levels via synbiotic/probiotic intervention due to regulatory homeostasis; thus, an increase in butyrate is most clinically meaningful among those who are depleted at baseline. Contextually, probiotic supplementation in healthy adults has shown limited and inconsistent effects on fecal butyrate, with only three studies measuring fecal butyrate reporting an increase [38]. Our results are consistent with previous findings that demonstrate 4-week supplementation with *Bifidobacterium bifidum* MIMBb75 in healthy adults led to enhanced butyrate levels only in individuals with lower baseline SCFA levels [18]. Similarly, 4-week supplementation with *Lactobacillus paracasei* DG in healthy adults also led to enhanced butyrate levels only in individuals with lower baseline butyrate levels [20]. In our study, the multi-species synbiotic led to a 66% increase in butyrate during the study among participants with low-baseline butyrate levels. This is an important finding given the critical role of butyrate in maintaining GI function (e.g., motility and barrier function), as previously described in the context of butyrate-producing bacteria. From a metabolic health perspective, butyrate regulates the secretion of intestinal GLP-1. Thus, it is interesting that we find a significant reduction in HbA1c levels observed in the synbiotic arm; however, these levels of HbA1c were within the normal range as would be anticipated for a healthy population without metabolic syndrome. This mechanistic interpretation is based on prior evidence rather than data generated in the present study as we did not measure intestinal GLP-1. Additionally, further studies are needed to determine whether the increases in butyrate levels lead to measurable clinical improvements in individuals with GI symptoms (e.g., bloating, constipation).

Fourth, an increase in abundance of synbiotic strains was significantly associated with a decrease in serum CRP levels, indicating that multi-species synbiotic supplementation may contribute to the regulation of low-grade systemic inflammation, even among healthy individuals. The reduction in CRP was most strongly associated with increases in *Bifidobacterium longum* and *Bifidobacterium infantis*, two species that have demonstrated anti-inflammatory effects by modulating immune cell activity and metabolite-mediated mechanisms. This multi-species synbiotic contains Bifidobacteria strains that have previously been shown to reduce inflammation in healthy individuals, supporting recovery and performance in athletes [39]. The impact of the multi-species synbiotic (−0.44 mg/L CRP) is complementary to decreases in CRP induced by lifestyle habits (e.g., diet, exercise). Specifically, in a meta-analysis of 43 studies with healthy individuals, exercise intervention induced a decrease in CRP (−0.59 mg/L) [40]. In general, less systemic inflammation is associated with less clinically relevant disease and less long-term system-wide organ damage. Overall, these data suggest this multi-species synbiotic is associated with lower sub-clinical serum CRP levels in healthy adults, which may be complementary to lifestyle changes.

Fifth, the multi-species synbiotic was well-tolerated with no reported adverse events. A comprehensive blood safety panel of blood and standard adverse event reporting was conducted. Overall, there were no clinically significant changes in vital signs (e.g., HR, BP) or safety blood markers (e.g., renal function, liver function).

A limitation of the trial is that dietary intake was assessed using self-reported food records, which are subject to recall/memory bias and possible underreporting. Participants were provided with both verbal and written instructions, used a standardized online dietary tracking tool, and were counseled to replicate their diet to minimize variability. Nevertheless, residual dietary variability may have influenced microbiome and metabolite outcomes, and more rigorous methods of diet control may strengthen future studies. Although participants were instructed to avoid live-culture foods and supplements, and the trial was randomized, residual dietary variability may still have influenced the outcomes. This trial was conducted in generally healthy adults; however, future research is needed, particularly in individuals with GI symptoms to determine whether the biological benefit demonstrated in this study leads to clinical improvements in GI outcomes. Furthermore, fecal calprotectin was not measured in this study, and future research may benefit from evaluating intestinal inflammation marker in addition to systemic measures such as CRP. Lastly, this mechanistic-focused trial had a relatively small sample size, accordingly larger clinical-focused trials may be warranted.

Despite this limitation, this clinical trial demonstrated that a multi-species synbiotic meaningfully impacted host biology important in GI function in healthy adults. Future research should explore the impact of this multi-species synbiotic on populations with GI symptoms and other populations with microbiome-mediated conditions. Specifically, given the mechanisms-of-action of this multi-species synbiotic and the underpinning biology of bloating, which is driven by gas-producing microbes, impaired motility, and/or visceral hypersensitivity, a clinical population experiencing bloating may be an ideal target for a future randomized controlled trial.

## 5. Conclusions

This randomized, placebo-controlled trial demonstrates that daily supplementation with a multi-species synbiotic rapidly and sustainably increased microbiome diversity and increased key native beneficial microbes. The synbiotic converted all participants into Urolithin A producers, leading to robust increases in UroA across all time points, enhanced fecal butyrate levels and enriched key butyrate-producing taxa (*Roseburia*, *Butyribacter*, and *Blautia*), and reduced systemic inflammation, as reflected by lower serum CRP levels. Collectively, these findings establish that this multi-species synbiotic is the first intervention shown to simultaneously increase both UroA and butyrate. Overall, the trial demonstrates that this multi-species synbiotic impacts key microbiome-derived metabolites, in turn, influencing critical biological functions that regulate gastrointestinal symptoms and systemic health.

## Figures and Tables

**Table 1:** Eligibility Criteria.

Category	Criteria
Inclusion	(1) Healthy males and females between the ages of 18 to 55. (2) BMI of 18.5–29.9 kg/m^2^. (3) Waist circumference <102 cm in males or <88 cm in females. (4) Healthy as determined by laboratory results, medical history, and physical exam. (5) Agreed to abstain from use of fermented foods or beverages with live bacteria or products containing active cultures for the duration of the study. (6) Agreed to refrain from intake of nonsteroidal anti-inflammatory drugs. (7) Agreed to refrain from using drugs and supplements containing aluminum, magnesium, sorbitol, and/or mannitol. (8) Agreed to comply with all study procedures. (9) Agreed to maintain current level of physical activity throughout the study. (10) Female participant is not of child-bearing potential, defined as females who have undergone a sterilization procedure (e.g., hysterectomy, bilateral tubal ligation, total endometrial ablation), or females of child-bearing potential must have a negative baseline urine pregnancy test and agree to use a medically approved method of birth control for the duration of the study. (11) Provided voluntary, written, informed consent to participate in the study.
Exclusion	(1) Women who are pregnant, breast feeding, or planning to become pregnant during the trial. (2) Allergy or sensitivity to the investigational product’s active or inactive ingredients. (3) Use of antibiotics or antifungals within three months prior to enrollment, including topical antibiotics or antifungals. (4) Clinically significant abnormal laboratory results at screening; (5) Use of proton pump inhibitors and H2-antagonists in the past 3 months. (6) Use of tobacco/nicotine products in the past 12 months. (7) Type I or type II diabetes mellitus or treatment with anti-diabetic medication. (8) Unstable metabolic diseases or chronic diseases. (9) Documented or self-reported current or pre-existing thyroid condition. (10) Unstable hypertension. (11) Current or history of any significant diseases of the gastrointestinal tract (including but not limited to inflammatory bowel disease and diverticulosis). (12) Use of implantable device such as a heart pacemaker. (13) Significant cardiovascular event in the past 6 months. (14) Major surgery, including abdominal, in the past 3 months or individuals who have planned surgery during the course of the trial. (15) Documented or self-reported autoimmune disease or an immune-compromised state. (16) Documented or self-reported HIV-, Hepatitis B-, and/or C-positive diagnosis. (17) History of or current diagnosis with kidney and/or liver diseases and/or serious infections, with the exception of symptom free kidney stones’ history for 6 months. (18) Self-reported medical or neuropsychological condition and/or cognitive impairment. (19) Self-reported blood/bleeding disorder. (20) Cancer in the five years prior to enrollment, except skin cancers completely excised with no chemotherapy or radiation with a follow up that is negative. (21) Clinically significant illness in the four weeks prior to randomization or screening. (22) Current use of any probiotic, prebiotic, and synbiotic product unless willing to undergo a 4-week washout prior to run-in period and abstain from consuming such products during the study. (23) Use of any cannabinoid products (including synthetics) within one month of study entry or during the study. (24) Alcohol or drug abuse within the last 12 months. (25) High alcohol intake (average of >2 standard drinks per day or >10 standard drinks per week). (26) Blood donation 30 days prior to screening, during the study, or a planned donation within 30 days of the last study visit. (27) Participation in other clinical research trials 30 days prior to screening.

**Table 2:** Participant Characteristics.

	All (*n* = 32)		DS-01 (*n* = 16)		Placebo (*n* = 16)	
	Mean	SD	Mean	SD	Mean	SD
Age (years)	33.6	9.1	37.0	9.3	34.9	10.4
Sex (% female)	63	NA	63	NA	63	NA
Height (cm)	168.9	10.6	171.9	7.8	167.5	11.2
Body Weight (kg)	69.2	11.0	69.7	8.6	69.8	11.6
BMI (kg/m^2^)	24.2	2.5	23.5	1.8	24.8	2.7

**Table 3:** Changes in safety markers from screening to end of study. Hba1c: Hemoglobin A1c; eGFR: estimated glomerular filtration rate; AST: Aspartate Aminotransferase; ALT: Alanine Aminotransferase; RBC: Red Blood Cell count; MCV: Mean Corpuscular Volume; MCH: Mean Corpuscular Hemoglobin; MCHC: Mean Corpuscular Hemoglobin Concentration; RDW: Red Cell Distribution Width; WBC: White Blood Cell; MPV: Mean Platelet Volume; PLA: Placebo.

Marker	Study Arm	Day 0	Day 91	*p*-Value	*p*-Value
		Mean ± SD	Mean ± SD	Within Arms	Between Arms
Bilirubin (µmol/L)	PLA	2.3 ± 0.5	2.1 ± 0.5	0.51	0.08 (Day 0)
	Synbiotic	2.0 ± 0.7	2.2 ± 0.5	0.07	0.71 (Day 91)
Creatinine (µmol/L)	PLA	75.9 ± 14.7	77.3 ± 17.1	0.48	0.52 (Day 0)
	Synbiotic	72.9 ± 8.7	75.3 ± 10.4	0.35	0.58 (Day 91)
Glucose (mmol/L)	PLA	5.0 ± 0.3	5.1 ± 0.2	0.78	0.69 (Day 0)
	Synbiotic	5.1 ± 0.4	5.1 ± 0.4	0.68	0.64 (Day 91)
HbA1c (%)	PLA	5.3 ± 0.3	5.2 ± 0.3	0.18	0.05 (Day 0)
	Synbiotic	5.5 ± 0.2	5.4 ± 0.2	0.03	0.10 (Day 91)
eGFR (mL/min/1.73 m^2^)	PLA	100.1 ± 16.6	98.1 ± 17.9	0.51	0.90 (Day 0)
	Synbiotic	100.8 ± 17.9	98.3 ± 14.0	0.07	0.90 (Day 91)
Sodium (mmol/L)	PLA	139.3 ± 1.8	140.0 ± 2.9	0.36	0.10 (Day 0)
	Synbiotic	140.6 ± 2.0	140.6 ± 1.7	0.97	0.45 (Day 91)
Potassium (mmol/L)	PLA	4.7 ± 0.3	4.5 ± 0.4	0.17	0.61 (Day 0)
	Synbiotic	4.7 ± 0.4	4.3 ± 0.3	0.01	0.07 (Day 91)
Chloride (mmol/L)	PLA	100.4 ± 1.5	103.6 ± 3.8	0.71	0.50 (Day 0)
	Synbiotic	104.0 ± 1.9	103.8 ± 1.6	0.81	0.89 (Day 91)
AST (U/L)	PLA	19.4 ± 5.9	20.6 ± 4.9	0.53	0.39 (Day 0)
	Synbiotic	17.9 ± 3.6	18.5 ± 4.1	0.51	0.41 (Day 91)
ALT (U/L)	PLA	17.0 ± 8.1	18.4 ± 6.9	0.58	0.73 (Day 0)
	Synbiotic	16.1 ± 6.6	17.8 ± 9.7	0.29	0.97 (Day 91)
Hemoglobin (g/L)	PLA	142.4 ± 10.4	140.2 ± 11.7	0.13	0.05 (Day 0)
	Synbiotic	134.4 ± 11.2	134.6 ± 11.0	0.98	0.17(Day 91)
Hematocrit (L/L)	PLA	0.43 ± 0.03	0.42 ± 0.3	0.02	0.06 (Day 0)
	Synbiotic	0.40 ± 0.0	0.40 ± 0.3	0.34	0.18 (Day 91)
RBC (×1021/L)	PLA	4.7 ± 0.3	4.6 ± 0.4	0.03	0.03 (Day 0)
	Synbiotic	4.4 ± 0.5	4.4 ± 0.4	0.14	0.06 (Day 91)
MCV (ft)	PLA	88.8 ± 4.8	90.0 ± 4.8	0.84	0.24 (Day 0)
	Synbiotic	91.6 ± 3.9	91.3 ± 4.0	0.89	0.22 (Day 91)
MCH (pg)	PLA	30.0 ± 1.6	30.5 ± 1.7	0.16	0.21 (Day 0)
	Synbiotic	30.7 ± 1.5	30.9 ± 1.6	0.24	0.27 (Day 91)
MCHC (g/L)	PLA	335.4 ± 5.6	337.9 ± 6.3	0.25	0.80 (Day 0)
	Synbiotic	334.8 ± 6.8	337.4 ± 7.4	0.23	0.84 (Day 91)
RDW (%)	PLA	13.1 ± 0.6	13.0 ± 0.7	0.45	0.41 (Day 0)
	Synbiotic	13.3 ± 0.7	13.4 ± 0.9	0.44	0.17(Day 91)
WBC (×10^9^/L)	PLA	1.7 ± 0.3	1.6 ± 0.3	0.13	0.06 (Day 0)
	Synbiotic	1.6 ± 0.2	1.6 ± 0.2	0.29	0.89 (Day 91)
Platelets (×10^9^/L)	PLA	239.9 ± 55.4	223.8 ± 55.3	0.09	0.76 (Day 0)
	Synbiotic	234.8 ± 42.3	234.1 ± 28.6	0.41	0.48 (Day 91)
MPV (fl)	PLA	8.7 ± 0.6	9.1 ± 0.7	0.02	0.07 (Day 0)
	Synbiotic	9.3 ± 1.0	9.3 ± 0.9	0.77	0.36 (Day 91)
Absolute Neutrophils (×10^9^/L)	PLA	1.2 ± 0.3	1.0 ± 0.3	0.11	0.13 (Day 0)
	Synbiotic	1.0 ± 0.3	1.0 ± 0.2	0.07	0.84 (Day 91)
Absolute Lymphocytes (×10^9^/L)	PLA	1.7 ± 0.5	1.6 ± 0.5	0.28	0.09 (Day 0)
	Synbiotic	1.4 ± 0.4	1.5 ± 0.4	0.29	0.72 (Day 91)
Absolute Monocytes (×10^9^/L)	PLA	0.4 ± 0.1	0.4 ± 0.1	0.95	0.61 (Day 0)
	Synbiotic	0.4 ± 0.1	0.4 ± 0.1	0.70	0.78 (Day 91)
Absolute Eosinophils (×10^9^/L)	PLA	0.2 ± 0.2	0.2 ± 0.2	0.09	0.09 (Day 0)
	Synbiotic	0.1 ± 0.1	0.1 ± 0.1	0.22	0.06 (Day 91)
Absolute Basophils (×10^9^/L)	PLA	0.01 ± 0.02	0.01 ± 0.02	1.00	0.60 (Day 0)
	Synbiotic	0.00 ± 0.02	0.02 ± 0.03	0.07	0.38 (Day 91)

## Data Availability

Data and statistical analyses are available for non-commercial scientific inquiry and/or educational if request and use does not violate IRB restrictions and/or research agreement terms.
